# The Relationship between Urban Vibrancy and Built Environment: An Empirical Study from an Emerging City in an Arid Region

**DOI:** 10.3390/ijerph18020525

**Published:** 2021-01-10

**Authors:** Runde Fu, Xinhuan Zhang, Degang Yang, Tianyi Cai, Yufang Zhang

**Affiliations:** 1 State Key Laboratory of Desert and Oasis Ecology, Xinjiang Institute of Ecology and Geography, Chinese Academy of Sciences, Urumqi 830011, China; furunde18@mails.ucas.ac.cn (R.F.); dgyang@ms.xjb.ac.cn (D.Y.); caitianyi14@mails.ucas.ac.cn (T.C.); zhangyf@ms.xjb.ac.cn (Y.Z.); 2 The Graduate School, University of Chinese Academy of Sciences, Beijing 100049, China

**Keywords:** urban vibrancy, built environment, influence, GWR, multi-source data, Urumqi

## Abstract

Creating a vital and lively urban environment is an inherent requirement of urban sustainable development, and understanding urban vibrancy is helpful for urban development policy making. The urban vibrancy theory needs more empirical supplementation and more evidence for the effect of the built environment on urban vibrancy. We use multisource urban spatial information data, including real-time population distribution (RPD) data and small catering business (SCB) data; quantitatively measure urban vibrancy; and build a comparative framework to explore the effect of the built environment on the urban vibrancy of a northwestern emerging city in China. The results demonstrate that the two urban vibrancy metrics present a spatial distribution pattern that is high in the south and low in the north areas of the city with significant spatial aggregation. Land-use intensity and diversity have strong positive effects on urban vibrancy but present a different pattern of effects on the two vibrancy measures. The influences on urban vibrancy of distance to the district center and distance to the nearest commercial complex are spatially complementary in the study area, and the effect of accessibility factors is weak. Our findings suggest that a somewhat cautious approach is required in the application of these classical planning theories to Urumqi.

## 1. Introduction

Urban studies always focus on temporal and spatial relationships between human activities and urban spatial entities. With urban land sprawl and rapid urbanization, the creation of vital and lively urban environments has attracted increasing attention. The challenge of urban expansion and pressure of urban renewal have led to critical reflections on urban planning and design. In the 1960s, from the social needs perspective, Jacobs criticized the urban design principle, which was not concerned with the natural features of cities, and then presented the concepts of urban diversity and urban vibrancy [1]. After that, many influential urbanists and scholars, such as Lynch and Montgomery, established a rich theoretical foundation for research on urban vibrancy [2]. As an important part of urban studies, urban vibrancy reflects human activities and human interactions with cities [3]. Urban vibrancy has been regarded as an essential element of urban quality of life that is connected with urban functions, urban form and socioeconomic activities [4,5,6]. Research on urban vibrancy is of great practical significance for urban quality improvement and sustainable urban planning and policy making.

Recently, urban vibrancy studies have attracted attention not only from urban designers but also from geographers and sociologists [7]. Researchers from multiple disciplines are commonly focusing on the inner mechanism of urban vibrancy and how to portray vibrancy [8,9,10]. Urban vibrancy can be regarded as a social process that is strongly related to the built environment [9]. Jacobs believed that vibrancy comes from the activities of people and the diversity of space, which largely depend on the morphological features of a city, land-use configurations and other built environment elements [10]. Many empirical studies have explored the relationship between the built environment and vibrancy, and some practical conclusions have been verified [11,12,13]. These practical conclusions are helpful to government decision making in urban planning. However, although some insightful efforts have been devoted to urban vibrancy research, difficulties and limitations remain. Divergence still exists in different research conclusions because of differences in vibrancy definitions and measurements. Moreover, there are still many questions in urban vibrancy studies that need to be settled. Two main issues have been raised: urban vibrancy portrayal and exploration of determinants [7,10]. Urban vibrancy has rich connotations, and different researchers hold different views on the subject. Such a situation has created difficulty in determining a good proxy to represent vibrancy. Previous studies have used various spatiotemporally tagged data, including field observations and interview data [13,14], Hand-held GPS tracking data [8], cellular signaling data [7,12], small catering business data [2,10] and point-of-interest (POI) data [15], to portray vibrancy. In fact, some of these data are extremely costly or limited by spatial scale, and few of them can clearly depict urban vibrancy. Similarly, built environment factors, such as density, mixed land use, typology and accessibility, which have been widely debated [2,16], are difficult to capture and quantify.

Many studies have used single-source urban data to portray the urban vibrancy [6,12,16]. Actually, a single source of urban data may reveal inaccurate spatial dynamics in one case of urban vibrancy [7], because they are accompanied by potential biases in penetrated population, spatial coverage, or sampling methods, etc., which may cause misunderstandings [17,18]. At the same time, the effects of determinants on different sourced urban vibrancies may be inconsistent. Moreover, even though a number of studies have highlighted the profound effect of mixed use [16], density [9] and accessibility [5] on urban vibrancy, some scholars have begun to question the effects of these indicators on vibrancy [10,11,12,19]. The effect of built environment indicators on urban vibrancy and the internal mechanism of urban vibrancy remain unclear [8].

Although the concept of urban vibrancy came out in the 1960s and has been widely accepted in urban planning and design, it was not until the early 2000s that these theories received much attention in China [11]. It is clear that the theory of urban vibrancy was proposed and developed based on the process of Western urbanization, and there are significant undeniable differences between Chinese and Western urban development [20]. In the past 20 years, China’s urbanization has been characterized by rapid and low-quality urban expansion [4]. Furthermore, there are huge differences in urban development across China, as different cities have indeterminate development stages and different construction situations, and the conclusions of an urban vibrancy evaluation might therefore be inconsistent [11]. Most recent studies of Chinese urban vibrancy have focused mainly on the developed and highly urbanized regions in eastern China, such as Shanghai [12], Shenzhen [7,9] and Wuhan [21]. Little attention has been paid to cities of the less developed interior regions in northwestern China, where the urbanization level is increasing rapidly; these areas have great development potential and urban renewal demand. Therefore, evidence from an emerging city with different stages of development is necessary to enrich urban vibrancy theory and provide validation from a new perspective. We select an emerging city, Urumqi, in northwestern China as our study region to apply and validate the classical urban vibrancy principles in a different city.

In this study, we use multisource urban spatial information data, including a new type of dataset, from different internet map service platforms to quantitatively measure the built environment effect. We use two urban vibrancy metrics, real-time population distribution data (RPD) data and small catering business (SCB) data, to build a comparative framework to explore influencing mechanisms. The effects of related built environmental factors, including diversity, accessibility, land-use intensity and location, are systematically explored with spatial statistical methods to determine the common built environment elements in the two types of vibrancy influence and thus to obtain a more robust conclusion.

The remainder of this article is organized as follows: Section 2 provides a literature review. Section 3 introduces the study area and lists the urban datasets and measurement methodology of vibrancy measures, independent variables, and regression models. Section 4 reports the analysis results. Section 5 offers a discussion and concluding remarks on this study.

## 2. Literature Review 

### 2.1. Urban Vibrancy and Measurement

Urbanization is social life supported by the agglomeration of “population size, density and heterogeneity” [22]. Urban vibrancy is the external representation of this kind of social life. Jane Jacobs proposed that urban vibrancy stems from people and their activities in a space. A vibrant street and neighborhood are able to attract many people and socioeconomic activities. Urban vibrancy can be considered as the intensity of people’s concentration [1]. Kevin Lynch proposed that vibrancy is the level of support for life and the requirements of ecology and human beings [3]. Montgomery noted that a vibrant urban area should be an open space that breeds high-density human activities [4,23]. Vibrancy is regarded as an essential element of urban quality of life, is closely associated with activity intensity and is an important characteristic of public spaces [6]. Ye Yu believed that urban vibrancy can be regarded as a social process closely related to the urban form, especially the physical entity of the built environment [22]. The foregoing viewpoints indicate that the key to generating vibrancy is having enough people and a good urban form.

How to measure the intensity of human activities is the key to quantify urban vibrancy. However, studies on urban vibrancy have long been subject to the difficulty of acquiring appropriate proxies and effective evaluation methods. Traditional research has always used field observation and interview data and survey data to depict vibrancy. For instance, Filion and Hammond measured neighborhood vibrancy by interviewing residents on how they utilize their surrounding environments in Waterloo [14]. Sung H and Lee S studied the applicability of Jacobs’ street vibrancy principle in Seoul based on survey data from interviews with residents of Seoul communities [24]. Ewing et al. conducted quantitative research on urban space construction and walkability by means of photographic recording [25]. These data sources are reasonable proxies for urban vibrancy, as such data directly record human activities, interactions, and living experiences [13]. However, obvious shortcomings exist. This type of data is limited by sample size, collection is extremely costly and time-consuming, and the data cannot be generalized to a vibrancy analysis over a large area. Moreover, some research has used Hand-held GPS tracking data to measure vibrancy [8]. Similarly, Hand-held GPS tracking data are usually available only at relatively small scales and are costly, with inherent low sampling rates and self-reporting biases [10].

Through recent advances in ICT (information and communication technology) and spatial orientation technologies, especially location-based service technologies, lifestyles have been undergoing great changes, and massive amounts of microscale spatiotemporal geographical open data are now available. Various spatiotemporally tagged data, including cellular signaling data, social media check-in data, point-of-interest (POI) data, smart-card records, taxi trajectory data, SCB data, and street view data, have been widely used in urban planning, population prediction [26,27], socioeconomic development [28,29], and urban vibrancy research [21], and even to infer individual personality [30]. Meanwhile, this new data environment provides the possibility of capturing human activities, rhythms, and preferences on a massive scale [31], enabling us to portray and quantify vibrancy much more easily and more accurately. Some pioneering works have used cellular signaling data to analyze vibrancy in communities [32], and geographical boundary detection has also been used [33]. Recently, researchers have used many kinds of spatiotemporally tagged data to evaluate urban vibrancy. De Nadai et al. derived a vibrancy metric from the density of mobile phone internet records and explored the vibrancy of six Italian cities [34]. Wu et al. took hourly social media check-in data as a proxy for urban vibrancy and explored the complex relationships with land-use configurations [6]. Wei Tu et al. indicated the urban vibrancy spatial patterns of Shenzhen by a comprehensive urban vibrancy metric formed by multisource data, including POIs, social media check-ins, and mobile phone records [7]. Ye Yu and Chang Xia et al. suggested that SCB can be an indicator of vibrancy because of the socioeconomic attributes of the attraction of pedestrian flows and urban activities [9,10]. There are some inherent advantages of SCB, even though they cannot reflect all aspects of urban vibrancy [9]. Places where small catering businesses are developed tend to be densely populated also attract walking, resting and other leisure activities. A small catering business with a vast consumer base and consumption potential is more flexible compared with large catering enterprises and department stores [10]. 

On a macro urban scale, the best indicator of activity intensity might be the number of active people per unit area. Nevertheless, although there are many types of data to choose from, few datasets can reflect real-time human mobility, and the real-time dynamic distribution of people is difficult to measure accurately on an urban scale [12]. With high rates of smartphone penetration, location-based service technology provides the possibility of tracking RPD on an urban scale. Many internet map service platforms, such as Baidu Maps, Amap, and Tencent Easygo, provide highly precise human mobility data. Among these platforms, Tencent Easygo, a thermal map of population distribution, is the most appropriate RPD tool based on the huge number of Tencent product registered users. Tencent Easygo provides a 25 m × 25 m spatial pattern of population distribution and real-time local pedestrian flow and congestion level [35,36]. Tencent Easygo has been applied in many Chinese urban studies investigating subjects such as urban functional area delineation [36], urban spatial structure analysis [37], commuting transport and job–housing balance [38]. With good data quality, Tencent Easygo data can provide excellent urban vibrancy metrics. However, few urban vibrancy studies have used Tencent Easygo data. Moreover, it is undeniable that multisource urban data may not reveal the same spatial patterns of urban vibrancy, causing potential biases [7]. This creates substantial difficulty in constructing an evaluation indicator of vibrancy in many types of urban big data. Hence, there is a need for a new perspective and comparative study.

### 2.2. Built Environment and Urban Vibrancy

Urban planning scholars and geographers have always been committed to forming a mechanism of urban vibrancy. Urban vibrancy depends on the agglomeration of people, and people’s necessary activities and autonomous activities are supported or blocked by the external environment. Consequently, urban vibrancy can be regarded as a social process that is strongly related to the built environment [9]. Many empirical studies have explored the relationship between the built environment and vibrancy, and some practical conclusions have been verified [11,12]. Many studies have found that built environment indicators, including diversity, suitable spatial scales, accessibility, and construction density, affect urban vibrancy [11,39]. Jacobs believed that vibrancy comes from the activities of people and the diversity of space, which largely depend on the morphological features of a city, land-use configurations and other built environment elements [10]. Montgomery inferred that a vital urban space needs mixed land use and diversity [16,18]. Wanshu Wu et al. confirmed that mixed land use, diversity, scale, old buildings, density and border vacuums are all connected to the vibrancy of Chinese high-density urban areas [12]. Chaogui Kang et al. found that activity, time, and space diversity are essential components of urban vibrancy [21]. Ye et al. reported that building typology and density are connected with significant positive effects on urban vibrancy [9]. 

However, several studies have drawn different conclusions about the effects of such factors as mixed land use and density on urban vibrancy, and some scholars have begun to question the effects of these indicators on vibrancy [10,11,12,19]. Chang Xia et al. found that although a significant positive spatial autocorrelation between land-use intensity and urban vibrancy exists, there are still local spatial mismatches which indicate that high density may not guarantee vibrancy [10]. Wanshu Wu et al. indicated that diversity will inhibit urban vibrancy during nonworking hours [12]. A comparative analysis of Beijing and Chengdu found that construction intensity indicators had different effects on the vibrancy of cities with different construction models and ages of buildings [11]. Furthermore, traffic congestion and noise, messy living conditions, air pollution and waste caused by high density, and old buildings cannot be ignored. These contradictions in the existing studies reflect an unclear and complex relationship between urban vibrancy and the built environment. In addition, for different cities with indeterminate development stages and different construction situations, the conclusions of urban vibrancy evaluations might be inconsistent. Previous studies of Chinese urban vibrancy have focused mostly on Chinese international metropolises such as Shanghai [12] and Shenzhen [6,7,8]. These international metropolises have entered the late stage of urbanization, with high population density, land-use intensity and sufficient urban facilities, and the speed of urban renewal has slowed. Nevertheless, few researchers have investigated northwestern emerging Chinese cities, such as Urumqi, which have tremendous development potential and urban renewal demand. Additionally, existing studies lack a multifaceted view of the commonalities and differences in how built environment elements influence vibrancy. Hence, there is a need for a comprehensive and comparative study. Consequently, it is meaningful to conduct empirical analysis of the effect of the built environment on urban vibrancy for emerging cities from multiple perspectives.

## 3. Data and Method 

### 3.1. Study Area

**Figure 1:**
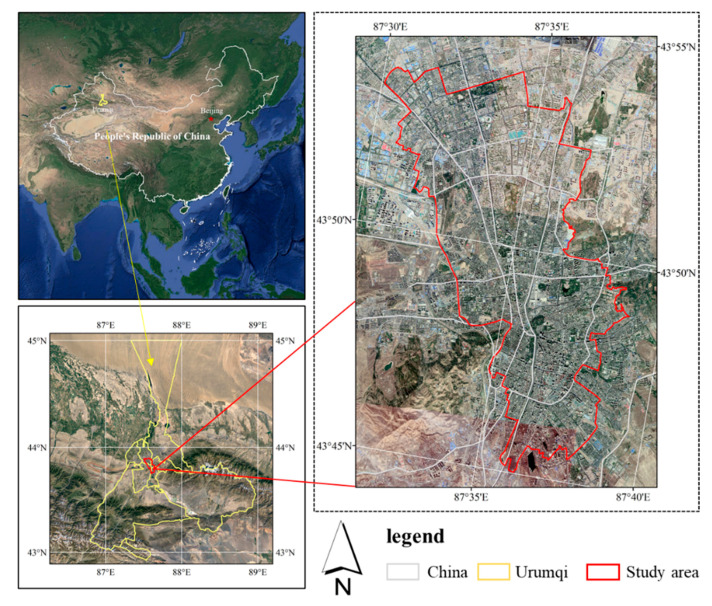
Study area: Urumqi, China (based on map sources: GS (2020)3183).

Urumqi (42°45′ to 44°08′ N, 86°37′ to 88°58′ E) is the capital and the political, economic, cultural and scientific and technological center of the Xinjiang Uyghur Autonomous Region. It covers approximately 14,216.3 km2 and has eight administrative districts: Tianshan District, Xinshi District, Shayibke District, Toutunhe District, Dabancheng District, Midong District, Shuimogou District and Urumqi County. In 2019, its GDP exceeded 341.3 billion yuan [40], ranking second among the cities of China’s five northwestern provinces. At the end of 2019, the number of permanent urban residents was 3.552 million. With disparate geographic conditions, the development and construction conditions of Urumqi are completely different from those of the popular cities of Chinese urban vibrancy research, such as Beijing, Shenzhen and Shanghai. As an oasis city in an arid region, Urumqi is one of the most important emerging cities in northwestern China and Central Asia. As one of the most important node cities of the Belt and Road initiative, Urumqi is an international Chinese trade center facing Central Asia, West Asia and Europe [41] with great potential for development and demand for urban renewal. To validate the impact of the built environment on urban vibrancy in Urumqi, we conduct a case study in the central urban area of the city (Figure 1), where most socioeconomic activity is concentrated. The analysis unit for studying vibrancy is very important [16]. The basic spatial analysis unit in this paper is a 300 m × 300 m grid, which is close to the average block size.

### 3.2. Data Sources and Measurement

#### 3.2.1. RPD Data

Most urban vibrancy studies have used mobile phone signaling data to represent real-time human mobility and distribution. However, the high costs, complex data processing flow, time lag and privacy issues make it difficult to obtain such data. Tencent Easygo is based on a large base of users of Tencent products, such as Tencent QQ (with 0.69 billion monthly active accounts in 2020), WeChat (1.15 billion monthly active accounts in 2019), and Tencent games (0.2 billion users) [37], and provides real-time locations of active users of Tencent products. Thus, it can reflect the spatial distribution of the population in the study area. Tencent Easygo data are relatively easy to obtain without encountering privacy issues or data qualification. Owing to its huge user base, it can provide a 25 m × 25 m spatial pattern of population distribution and real-time local pedestrian flow and congestion level, which highly precisely reflect human mobility. Some researchers have noted that urban activities are distinctly differentiated at particular times of day, on different days of the week, and between weekdays and weekends [42]. Tencent Easygo data can also take into account the overall characteristics of people’s behavior in different time periods.

Hence, we use workday data (21 May 2020–22 May 2020) and weekend data (30 May 2020–31 May 2020) at the same time to calculate local vibrancy. (Although China was struck by the COVID-19 epidemic at the beginning of 2020, and its social economy was greatly impacted, by the end of March, the epidemic in Urumqi had been effectively brought under control, and the normal social order basically resumed in May after scientific prevention and control measures and sustained economic stabilization policies.) The data were obtained by the web crawler at one-hour intervals over a time range of 7 am to 23 pm, the main time frame within which urban activities take place. The data records included grid point value, longitude, latitude and acquisition time. 

#### 3.2.2. SCB Data

SCB data have been widely accepted as an indicator of the attractiveness of urban places because of the socioeconomic attributes of the attraction of pedestrian flows and urban activities [9,10,19].

We used SCB data acquired in April 2020 from registered business information and check-in records in Dazhong-Dianping (https://www.dianping.com/), an aggregated social media tool used to rate restaurants and other service industry companies in China [43]. In total, we collected 19,170 small catering POIs from Dianping.com with a total of 2,331,357 user comments. What needs illustration is that Dianping.com use a standard of classification that is different from Amap, so the catering POIs from Dianping.com and Amap are not equal in quantity.

#### 3.2.3. POIs

POIs represent a much finer-grained picture of land use and are good proxies for mixed land uses in contrast to conventional land-use data [16,44]. In this study, the POI data were collected from the Amap open platform (https://lbs.amap.com/), which provides many geographic open data and data analysis APIs. Amap is one of China’s most popular map navigation applications and map search engines. The original data contain 18 types of facilities. Through the data cleansing process, we identified 15 POI types (Table 1) that are closely related to the social economy: motor vehicle services, catering services, shopping services, life services, sports and leisure services, medical and health care services, accommodation services, scenic spots, business residences, government agencies and social organizations, science and education and cultural services, transportation facilities services, financial insurance services, companies, and public facilities. Ultimately, we obtained a total of 60,363 POIs in our study area in April 2020. 

### 3.3. Variables and Methods

**Figure 2:**
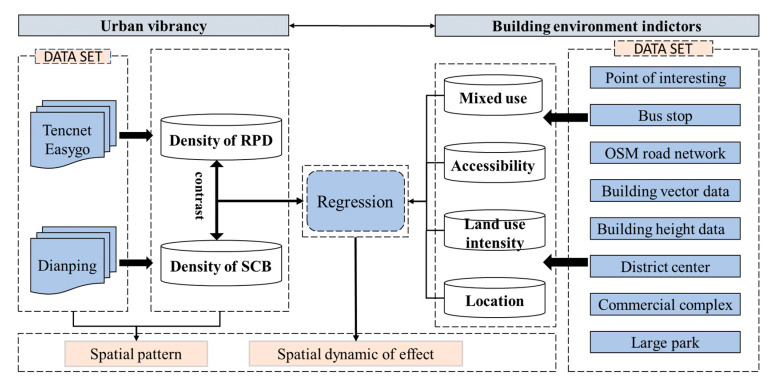
Research framework.

In this study, we use a comparative framework to explore the effects of built environmental factors on urban vibrancy. As shown in Figure 2, first, we choose two urban vibrancy metrics and build an index system of the built environment from four aspects based on multisource big data. Second, the spatial distribution patterns of RPD-derived vibrancy and SCB-derived vibrancy are investigated based on kernel density estimation (KDE). Because the original data is discontinuous point data, KDE could help us understand the overall spatial characteristics of urban vibrancy in the study area by resampling the data to a continuous smooth surface. Finally, we model the relationship between associated factors and urban vibrancy with geographical weighted regression (GWR) while exploring the significantly associated factors and mapping the spatial dynamics of their effects on urban vibrancy.

#### 3.3.1. Dependent Variables

KDE is conducted to reveal the spatial patterns of human activities from spatial and temporal perspectives. Through the process of interpolating discrete point or line data, KDE creates a smooth surface, which helps us analyze hot spots, estimate intensity and visualize the distribution of spatial features [45,46]. In this paper, KDE is used to transform discrete point data into continuous raster surfaces. The equation is as follows:

(1) $$f(s)=\sum_{i=1}^{n}\frac{1}{h^{2}}k(\frac{d_{is}}{h})$$

where *h* is the bandwidth, $d_{is}$ is the distance between point *i* and point *s*, and *k* is the space weight function.

For RPD data, we use ArcGIS to divide the research area into a 300 m × 300 m grid cells, which is consistent with the size of the study unit, and use the KDE tool to calculate the density value per hour. Weekend and workday sample data are integrated. Then, we obtain a mean density value for each cell. Finally, raster data are mapped to a vector grid. We use this mean value as the first vibrancy value. The higher the average value, the greater the overall heat of the region, and the more active the population. Similarly, for SCB data, taking user comments as weights, we use the KDE tool to calculate the Dianping density value for each grid, which is the second vibrancy value.

#### 3.3.2. Associated Factors

**Diversity:** The entropy function is used to measure mixed use (*MU*) in most research. However, some studies have found that entropy has limitations in mixed land-use measurements [20]. The TF-IDF algorithm is a commonly used term weighting method in information retrieval systems [47]. It is a statistics-based method that has been widely used in information retrieval and text mining. The kernel of its algorithm is the contribution rate of identifying categories. In this study, we use the TF-IDF algorithm combined with the Simpson index to calculate the mixed land-use indicator. This method considers the global distribution of a single type of POI to significantly improve the accuracy of the results. The mixed land use can be calculated as follows:

(2) $$F_{i}=\frac{n_{i,j}}{\sum_{k}n_{k,j}}$$

(3) $$T_{i}=\frac{\left|D\right|}{\left|\left\{j:t_{i}\in d_{j}\right\}\right|}$$

(4) $$A_{ij}=F_{i}\times T_{i}$$

where $F_{i}$ is the normalization of type i POI distribution, which prevents the algorithm from favoring the grid with a large number of POIs; *n_i,j_* is the frequency of type *i* POIs in the grid; $\underset{k}{\sum }n_{k,j}$ is the total number of all POI categories in the grid; D is the total number of grids; $\left\{j:t_{i}\in d_{j}\right\}$ is the number of grids with type *i* POIs; *T_i_* reflects the universal importance of type i POIs; and *A_ij_* is the real contribution of each type of POI in the grid. Then, we use the Simpson index to calculate the diversity value. The Simpson index, originally developed in the field of biology, models the probability that two randomly selected individuals will be from the same category. The formula is as follows:

(5) $$simpson=1-\sum_{i=1}^{s}p_{i}{}^{2}$$

where s is the number of POI types in the grid and $p_{i}$ is the proportion of type i POI contributions to the sum value of all types. This computing method allows us to obtain a more accurate *MU* index than using the POI category number to calculate the Simpson index directly.

**Accessibility:** Accessibility is an important part of built environment measurements. We use the road intersection density index (*Den_intersection*) and mean distance to nearest bus station (*Mdist_NBS*) to represent accessibility. OpenStreetMap (OSM) data for major cities in China have always been used in urban studies because of their good quality [9]. Our road network data are downloaded from OSMNX, a Python package to retrieve, model, analyze, and visualize street networks from OSM (https://geoffboeing.com/2016/11/osmnx-python-street-networks/). We extract all road intersections of the study area, count the number of intersections in each grid and calculate the intersection density. We calculate every distance between a building and the nearest bus station and then obtain the zonal statistical mean value.

**Land-use intensity:** To measure land-use intensity, we use building vector data (including building height) acquired from Baidu Maps to calculate the floor-area ratio (*FAR*), building coverage ratio (*BCR*) and average building height (*ABH*). The *FAR* is an important indicator that is positively related to construction intensity. The equation is as follows:

(6) $$FAR_{i}=\frac{Fa_{i}}{s_{i}}$$

The *BCR* is used in this study to measure the ground construction strength. It is the ratio of the built-up area in the grid to the area of the grid, as shown in the following:

(7) $$BCR_{i}=\frac{Bc_{i}}{s_{i}}$$

where $S_{i}$ denotes the area of grid i (m^2^), $Fa_{i}$ is the gross floor area of grid *i*, and $Bc_{i}$ is the building footprint of grid *i* (m^2^).

**Location:** Regional advantages are also important for attracting human activities and interactions. We take the distance to the district center (*Mdis_DC*), the mean distance of the nearest commercial complex (*Mdis_NCC*) and the mean distance of the nearest large park (*Mdist largepark*) into consideration to verify whether location is important. Table 2 shows descriptive statistics for all variables. 

#### 3.3.3. Spatial Autocorrelation

To demonstrate the spatial distribution pattern of vibrancy, the global Moran’s I was used to calculate the potential interdependence of vibrancy between grids [48]. The equation is as follows (Equation (8)):

(8) $$I=\frac{n}{\sum_{i=1}^{n}\sum_{j=1}^{n}w_{i,j}}\times \frac{\sum_{i=1}^{n}\sum_{j=1}^{n}w_{i,j}z_{i}z_{j}}{\sum_{j=1}^{n}z_{i}{}^{2}}$$

where *n* is the number of grids, *w_i,j_* is the spatial weight between the *i*th and *j*th grids, and *z_i_* is the deviation between the vibrancy of the *i*th grid and the mean value. The Moran’s *I* value is between −1 and 1. A value greater than 0 means a positive autocorrelation, while a value less than 0 means negative autocorrelation. Then, the significance test of the *Z* value of Moran’s I value was conducted to determine whether there is a spatial autocorrelation (Equation (9)):

(9) $$Z(I)=\frac{\left[I-E(I)\right]}{\sqrt{Var(I)}}$$

where *Z*(*I*) is the significance level of the global Moran’s *I*, *E*(*I*) is the mathematical expectation of Moran’s *I* and Var(*I*) is the variance in Moran’s *I*.

#### 3.3.4. GWR

Global regression analysis, such as ordinary least square (OLS) regression, requires that the estimated parameters have global and stationary properties. However, spatial data always have spatial autocorrelation or spatial heterogeneity, which makes it difficult to meet the assumptions and requirements of the OLS method. In comparison, GWR, which embeds spatial structure in a regression model, is more suitable for analyzing spatial data. In this study, GWR models were used to investigate the relationship between built environment factors and urban vibrancy and to explore the spatial heterogeneity of influence in consideration of spatial autocorrelation. The existence of spatial heterogeneity emphasizes the local characteristics of spatial-process interaction; thus, the global model does not work. As a nonparametric local weighted regression, GWR solves the spatial nonstationarity problem that cannot be solved by traditional OLS regression. In this study, the Gaussian kernel function is used to model geographic weights. We used adaptive kernel bandwidth in this study, because sample density varies over the study area. The optimal kernel bandwidth is set according to the corrected Akaike information criterion (AICc) as described in Fortheringham et al. [49]. The AIC criterion method more easily avoids the overfitting problem; the selected optimal model is often more effective. The dependent variable is the human activities intensity, while the independent variables are the built environment factors described above.

(10) $$y_{i}=\beta_{0}(u_{i},v_{i})+\sum_{k}\beta_{k}(u_{i},v_{i})X_{ik}+\varepsilon_{i}$$

where ($u_{i},v_{i}$) are the space coordinates of sample *i*, $\beta_{k}\left(u_{i},v_{i}\right)$ is the value of continuous function *β_k_*(*u*,*v*) at sample *i*, $X_{ik}$ are independent variables, and $\varepsilon_{i}$ is a random error term. 

## 4. Results

### 4.1. Spatial Distribution Pattern of Urban Vibrancy

**Figure 3:**
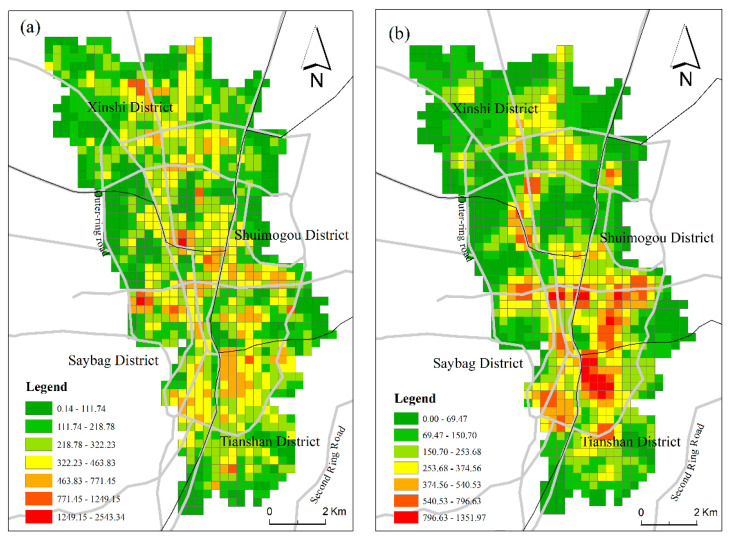
Spatial patterns of urban vibrancy in Urumqi: (**a**) RPD derived vibrancy; (**b**) SCB derived vibrancy.

Figure 3 shows the spatial distribution patterns, as indicated by two datasets, of urban vibrancy for Urumqi. The results reveal the following spatial patterns of urban vibrancy. Both RPD-derived vibrancy and SCB-derived vibrancy demonstrated differentiated spatial patterns of urban vibrancy in the central urban area of Urumqi, and both manifested relatively consistent spatial distribution patterns of vibrancy. The two urban vibrancy patterns demonstrated that most grids with high vibrancy were located inside the outer ring road, and the vibrancy of the south district was significantly higher than that of the north district. However, differences remained. RPD-derived vibrancy was more evenly distributed, yet SCB-derived vibrancy was more concentrated, and the high-value grids presented a more obvious dispersion distribution. From an administrative division perspective, vibrancy in Tianshan District and Shaybak District was higher than that in Xinshi District and Shuimogou District.

Further spatial autocorrelation analysis was performed on two vibrancy levels. Global Moran’s I was used to calculate the potential interdependence of urban vibrancy in the central urban area of Urumqi. The results in Table 3 illustrate that both vibrancy metrics demonstrated significant positive spatial autocorrelation phenomena. The results of Moran’s I were 0.444562 and 0.728335, respectively, and given the z-scores of 28.469256 and 46.12009, respectively, there was a less than 1% likelihood that this clustered pattern could be the result of random chance. SCB data manifested a higher spatial autocorrelation than RPD data. On the whole, general consistency was derived from the urban vibrancy metrics using two different data sources, and areas with similar vibrancy values tended to be spatially oriented with centralized distributions.

### 4.2. Results of Regression Model

First, we used a stepwise multiple regression model to calculate the overall difference characteristics of the influence of built environment factors on urban vibrancy and selected variables of significance. The analysis results are reported in Table 4. The RPD data-derived vibrancy explain only 30.7% of the total variation. The built environment factor has a better interpretation of the vibrancy based on SCB data, and the adjusted R^2^ in model 2 is 0.434. The results of the stepwise regression showed that the nine elements of the built environment are correlated with the urban vibrancy of Urumqi, and the significance of all factors is at the level of 0.05. Although the two vibrancy metrics corresponding to the impact of the built environment elements are not completely consistent, some consistent conclusions were obtained, verifying previous research conclusions. *MU*, *Mdis_DC*, *Mdis_NCC* and *FAR* are significantly associated with the two derived vibrancy results. *MU* and *FAR* have a strong positive effect on urban vibrancy. *Mdis_NCC* has the strongest negative effect, and the location indicator *Mdis_DC* has a weak negative effect.

The effects of the other elements were significant for only one vibrancy. *Mdist_NBS* has a weak negative effect on urban vibrancy based on RPD, and bus station density has a positive effect on urban vibrancy based on SCB. As a consequence, the results of the regression indicate that vibrancy occurs near bus stops, and the density of bus stops has an effect on Urumqi urban vibrancy. *Mdis_largepark* has a weak negative effect on vibrancy, which differs from the conclusions of previous research. *ABH* has a positive effect on RPD-derived vibrancy. Through a stepwise multiple regression model, we obtained two correct models and acquired variables of significance that will be used in GWR.

We retained factors with VIF values below 10 and those that exert significant effects on the independent variables from the results of stepwise multiple linear regression. Then, two GWR models were constructed for the two kinds of vibrancy to deconstruct the local characteristics of the effects of built environment factors, analyze the spatial heterogeneity of these influencing factors, and seek their universal characteristics. Table 5 shows the results of the two geographically weighted regression models, and both optimal bandwidths are 615. Through the calculation of GWR, the explanatory ability of the model is significantly improved. When the GWR component and the autoregressive operator are included, the adjusted R^2^ improves to 0.331 and 0.636, respectively. The interpretation of built environment elements for RPD-based urban vibrancy is still low. To facilitate analysis and comparison, we collated the regression coefficients of the calculation results and selected five statistical items of the regression coefficients: minimum, maximum, median, mean and standard deviation.

The different results of the regression coefficients show that the influence degree and action trend of each built environment factor are different in different grids. The results of the two models clearly indicate that the spatial effect of the nine influencing factors shows significant spatial differentiation. In the two models, the influence of *FAR* on the possessive grid is positive. All *Den_busstop* effects are positive in model 2. There are positive and negative differences in the effects of the other factors, from which we can draw some interesting conclusions. The median coefficients of *MU*, *ABH* in model 1, and *Den_busstop* in model 2 are positive numbers, meaning that these factors can stimulate vibrancy in most instances. The rest of the variables have a negative median, which means that these factors have a more negative effect on Urumqi urban vibrancy. In model 2, the influence of the built environment factors has greater spatial differentiation because the standard deviation of the coefficient is larger in model 2 than in model 1.

**Figure 4:**
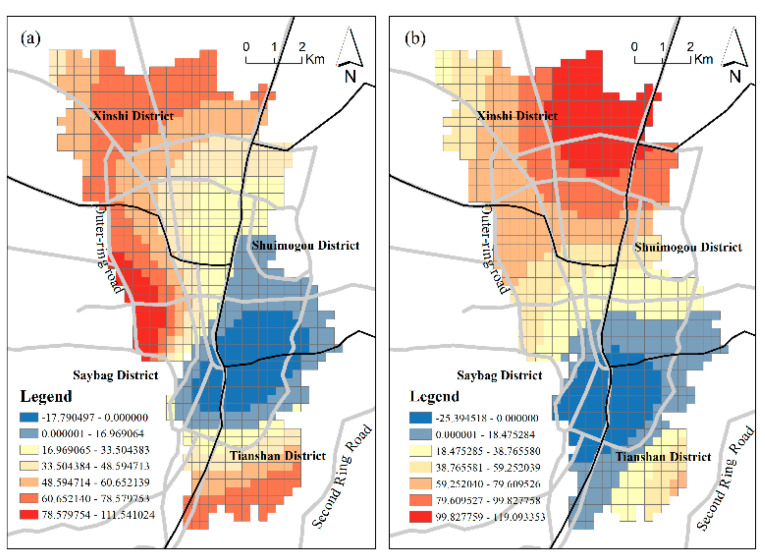
The coefficient of MU; (**a**) Model 1; (**b**) Model 2.

**Diversity:** The diversity of regional functions is represented mainly by mixed land use based on POI. We obtained an optimized *MU* index through the TF-IDF algorithm. In terms of the regression coefficient, mixed land use has a significant positive effect on urban vibrancy in most positions. The mean values of the regression parameters fitted by the two GWR models are all positive, and the two absolute values are both in second place. This verifies the positive effect of *MU* in stimulating regional vibrancy to a certain extent. Figure 4 shows the spatial distribution of the coefficients of *MU*, demonstrating different influences on vibrancy in different grids. However, the *MU* coefficient clearly showed a significant negative influence on some areas. The areas with negative effects are all distributed in the south urban area with a long history of development, which deviates from the traditional concept of mixed land use enhancing vibrancy. This area is located in the boundary area of Shuimogou District, Tianshan District and Saybag District, which is the old urban area of Urumqi with a complex diversity of functional space and the highest area of urban vibrancy. These abnormalities may occur for the following reasons: (1) As a result of a longer old city development time and a long history of urban planning, although the mixing degree of functional space due to natural selection is high, it may cause disorder and unbalanced space. Some functions are mismatched, which results in a vibrancy decrease. (2) The influence of other factors, such as *FAR*, masks that of *MU*.

**Accessibility:** Accessibility is an indicator commonly used to assess vibrancy [50,51]. Accessibility variables include *Mdist_NBS* and *Den_busstop*. *Mdist_NBS* has a weak negative influence on RPD-derived vibrancy, and *Den_busstop* is significant in model 2 and shows a weak positive effect. On the whole, the action intensity of accessibility elements is weak. The standard deviation (0.09976 and 0.14587) of these two coefficients is small, which indicates that there is little spatial difference in accessibility influence. This may be related to the selected study area, the central urban area of Urumqi, where accessibility is inherently good and public transport has full coverage. Consequently, the effect of accessibility factors showed small spatial differences. 

**Figure 5:**
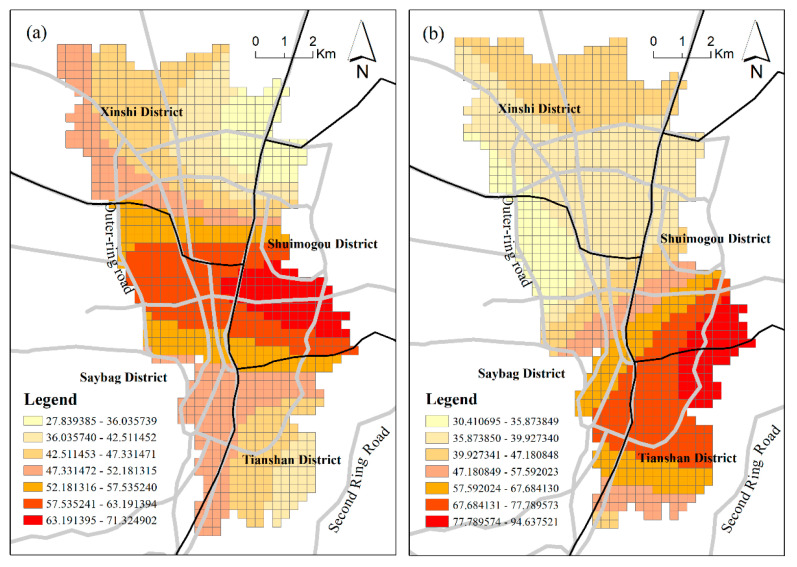
The coefficient of FAR: (**a**) Model 1; (**b**) Model 2.

**Land-use intensity:***FAR* is the built environment factor with the strongest positive effect on the urban vibrancy of Urumqi. The mean values of the coefficients in the two models are 49.63042 and 49.83, respectively, which is consistent with the conclusions of many previous studies [5,20]. FAR is an important indicator of land-use intensity. The greater the intensity of land use, the more social and economic activities will occur, promoting urban vibrancy. Thus, development density has positive effects on inducing vibrancy. The regression results (Table 5) indicate that the standard deviations in the two models are both small, which means the spatial differentiation of the *FAR* effect is small. However, a difference remains. The influences of *FAR* on RPD-derived vibrancy display a “sandwich” pattern (Figure 5a) with a high value in the central area and a low value on the periphery. Additionally, *FAR* presents a north–south divide in the influence of SCB-derived vibrancy. In the southern part of the study area, the land-use intensity has a greater stimulating effect on vibrancy, while in the northern part, due to the short development period, the land-use potential needs to be improved, and the impact on vibrancy is less. This finding demonstrates that northern Urumqi needs further strengthened urban construction, as more buildings attract additional human activities and interactions, thus increasing urban vibrancy.

**Figure 6:**
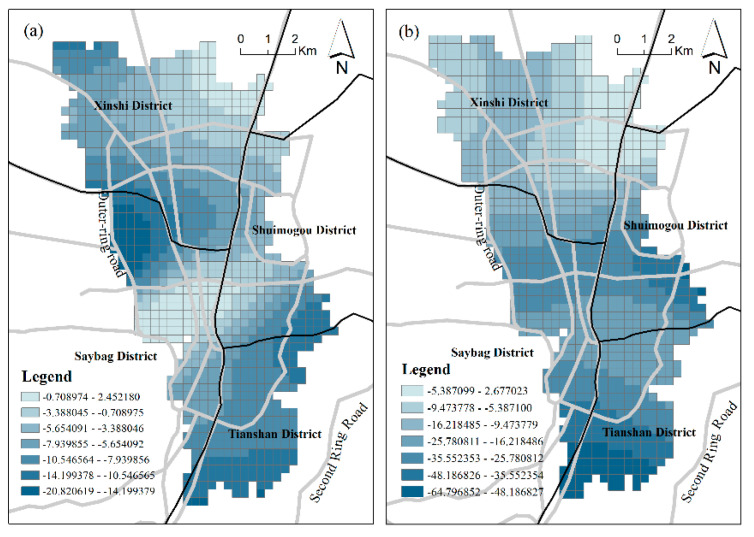
The coefficient of *Mdis_DC*: (**a**) Model 1; (**b**) Model 2.

**Location:** Location elements map to advantages of spatial position, such as shopping convenience. We selected three indicators, *Mdis_DC*, *Mdis_NLG*, and *Mdis_NCC*, to analyze the different spatial effects. The mean distance from the district center (*Mdis_DC*) has a significant negative influence on urban vibrancy, and the degree of influence varies with the change in position. The spatial patterns of the *Mdis_DC* effect are different for RPD-derived vibrancy and SCB-derived vibrancy, as shown in Figure 6. The negative effects of *Mdis_DC* on RPD-derived vibrancy are much higher at the margins of the study area. This demonstrates a trend of RPD vibrancy decreasing with distance from the district center and is consistent with a geographical decay function. The negative effect of *Mdis_DC* on SCB-derived vibrancy reveals a trend of higher negative effect in the southern area and lower negative effect in the northern area. On the whole, the negative effect of *Mdis_DC* diffuses outward. The further from the district center, the greater the impact.

**Figure 7:**
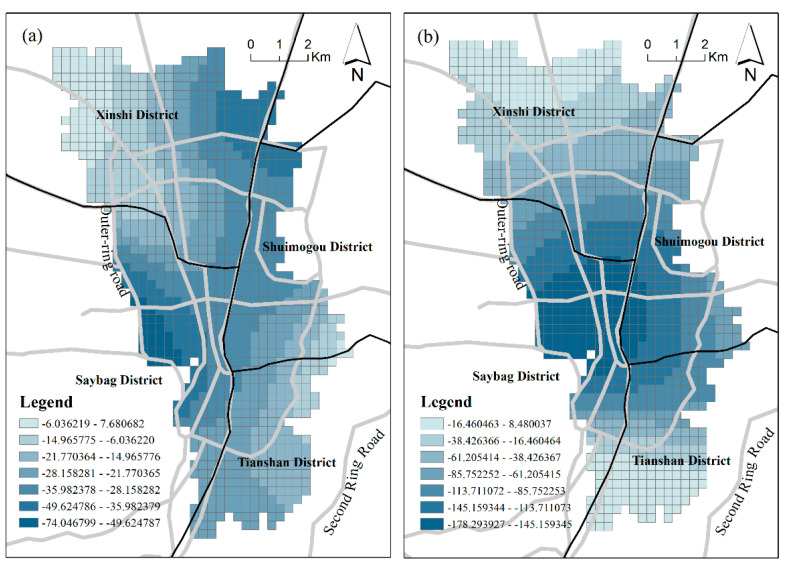
The coefficient of *Mdis_NCC*: (**a**) Model 1; (**b**) Model 2.

The commercial district is the most active gathering area for all kinds of economic activities. These economic activities can effectively attract large numbers of people and socioeconomic activities. Therefore, we believe that the area surrounding the commercial district can generally stimulate the vibrancy of the city. The calculation results of the GWR model show that the negative effect of *Mdis_NCC* on the two vibrancy metrics is the largest of all the factors, indicating that the development of the commercial district has a significant stimulating effect on the vibrancy of the region and that urban vibrancy can be significantly improved in places where there are large commercial blocks. The effect of *Mdis_NCC* has the largest spatial difference among all the factors, and the coefficient standard deviations of both models are large. The two vibrancy levels are negatively correlated with this factor, indicating the advantage of business centers in attracting vibrancy and the advantage of absorbing vibrancy. As shown in Figure 7, the *Mdis_NCC* effect has different spatial patterns in the two vibrancy metrics. The influences of *Mdis_NCC* on RPD-based vibrancy show a pattern extending northwest and southeast, while its influences on SCB-based vibrancy show a central-peripheral mode. Nevertheless, there are still similarities between them. The effects of *Mdis_NCC* occur much more within the inner ring line because of the concentration of commercial complexes in the central area of Urumqi. In other words, in the central study area, the negative effect of *Mdis_NCC* is stronger. This complements the influence of *Mdis_DC,* which is much higher at the margins of the study area.

Many studies have found that the effect of parks depends on their location and quality or that they have no significant effect on urban vibrancy [1,52,53]. The regression results demonstrate that *Mdis_largepark* has a weak negative influence on SCB-derived vibrancy. The reason may be related to the specific natural conditions in arid areas. As Urumqi is at the heart of the arid region of the Eurasian continent, there is no doubt that large green space is a valuable resource for the city. When people pursue this kind of resource, social and economic activities will be attracted to green spaces.

## 5. Discussion

Although the theory of urban vibrancy has been widely discussed, these conclusions need to be viewed dialectically against different urban development backgrounds. We chose an emerging city with a unique arid natural environment as the study area and explored the application prospects of urban vibrancy theory with new research objects. Many previous studies have emphasized the positive effects of density and diversity [9,10,11,12]. They follow Jacobs’s view that dense urban forms have more vibrancy than open urban forms [1,18,54], and the MU of a region in urban construction enhances the social bonds of group behavior within the region [5]. The results of our regression analysis show that in some cases, diversity may also lead to the disappearance of urban vibrancy, and excessive land-use intensity may suppress the creation of urban vibrancy [11]. Due to the different development stages of the city, commercial activities are very attractive to the vibrancy of Urumqi, which differs from the conclusions of studies based on cities in more developed regions in China. Therefore, future policies need to breed vibrancy, especially in north Urumqi, which will benefit well-being in this city. Future urban construction should further increase the intensity of land use and the degree of mixed functions in north Urumqi. Furthermore, it should give play to the advantages of driving vibrancy of commercial functions, and improve the commercial investment environment and traffic construction in northern districts. In the southern district, future development should prevent the disorderly development of the city, control the construction intensity, further transform and optimize the street environment, and try to solve the traffic congestion. 

Recently, rich multisource urban big data have provided a better opportunity to depict urban details and human behavior [9]. We used a new descriptive variable of urban vibrancy and proved its usability. Tencent Easygo reflects real-time population spatial distribution, which can profoundly reflect dynamic changes in vibrancy [36]. The analysis results show that the urban vibrancy mode has some similarities with the vibrancy model based on SCB data. By comparing datasets from previous studies, cellular signaling data [34], and social media check-in data [6], we provide a new data perspective that enriches the urban vibrancy characterization approach. In addition, some new methods are used to quantify the elements of the built environment. A comparative analysis framework is used to integrate the two dynamic influence mechanisms. Obviously, this analysis method can be applied to other cities. The spatial distribution pattern of urban vibrancy can be identified through high-resolution spatial data from multiple perspectives, which can provide comparison and correction for urban managers’ cognition. Secondly, through identifying the spatial mechanism of urban vibrancy, it can provide targeted reference measures based on the current development condition. In addition, we offer a supplement to the theory of urban vibrancy and provide relevant information for the government to optimize urban design and construction and pursue efficient approaches to promoting city vibrancy. 

However, there are some deficiencies in our research. First, RPD data can be used to present urban vibrancy effectively, but Tencent Easygo could not cover people of all ages, such as children and the aged, because of low smartphone penetration in these two age groups. Furthermore, due to the RPD coming out from human behavior that is hard to explain solely by built environmental factors, the RPD model has a relatively low R^2^. Second, limited by the availability of data, there is a lack of discussion of the effects of socioeconomic environmental factors, such as population and employment rates, on urban vibrancy. In addition, different urban vibrancy metrics do not completely match each other because of differences in the generation and collection processes. The different characteristics of the two kinds of vibrancy indicate that a single data source may depict an incomplete spatial pattern of urban vibrancy [9]. Therefore, future studies could collect more urban datasets related to urban vibrancy to construct a comprehensive vibrancy index. Cellular signaling data, social media check-in data, smart-card records, and bike-sharing and taxi trajectory data are needed to estimate population distribution and mobility more accurately. Additionally, limited by our research framework, we did not consider time diversity, although studies have shown that time diversity has a strong association with urban vibrancy [21]. Future studies should strengthen the collection and acquisition of long-term continuous data to dynamically monitor and assess the relationship between the built environment and urban vibrancy. 

## 6. Conclusions

Urban vibrancy is a social process that depends on the aggregation of people and the physical substance of the city in the complex urban system. It is mainly about expressing the ability of a region to absorb economic and social activities. This paper attempts to clarify the influence mechanism and spatial heterogeneity of urban built environment elements on Urumqi’s urban vibrancy by constructing two comparative analytical frameworks of vibrancy. We obtained some conclusions consistent with those of previous studies as well as specific conclusions with regional characteristics.

At first, despite some differences between our conclusions and classical theories, this study found empirical evidence that the built environment plays a significant role in promoting urban vibrancy in this arid region city of China. The urban vibrancy of Urumqi presents a spatial distribution pattern that is high in the south and low in the north. There is a significant spatial spillover effect of urban vibrancy, and a high-vibrancy area promotes vibrancy in the surrounding areas. The regression results show that the urban vibrancy of Urumqi is significantly correlated with the urban built environment, but the effects of various factors are not consistent with the classical cognition. FAR has a strong positive effect on urban vibrancy and presents a different pattern of effect on the two vibrancy metrics. The influence of FAR displays a “sandwich” pattern for RPD-derived vibrancy and a north–south divide for SCB-derived vibrancy. Mixed land use has a strong positive effect on urban vibrancy in most study areas but presents a negative effect in some places. On the whole, the influence of accessibility factors is weak, which might be related to the selected study area. As a valuable resource for Urumqi, distance to large parks and green spaces has a weak negative influence on vibrancy. The distance to the district center had the greatest influence on the vibrancy of the marginal region, while the distance to the commercial complex had the strongest influence on the vibrancy of the central urban area. These findings suggest that a somewhat cautious approach is required in the application of these classical planning theories to Urumqi.

## Figures and Tables

**Table 1:** Descriptive statistics of variables.

Category	Frequency	Percentage
motor vehicle services	2427	4.02%
catering services	8459	14.01%
shopping services	15,330	25.40%
life services	11,223	18.59%
sports and leisure services	1181	1.96%
medical and health care services	3476	5.76%
accommodation services	1230	2.04%
scenic spots	164	0.27%
business residences	3384	5.61%
government agencies and social organizations	2720	4.51%
science education and cultural services	2835	4.70%
transportation facilities services	1974	3.27%
financial insurance services	1642	2.72%
companies	3876	6.42%
public facilities	439	0.73%

**Table 2:** Descriptive statistics of variables.

Variable		Min	Mean	Max	Std
Real-time population distribution (RPD)		0.142	254.506	2543.340	190.986
Small catering business (SCB)		0.000	196.474	1351.980	189.330
Diversity	*MU*	0.000	0.708	0.917	0.246
Accessibility	*Mdist_NBS*	36.002	197.554	828.430	110.021
	*Den_busstop*	0.000	23.844	222.222	34.509
	*Den_intersection*	0.000	43.116	733.000	63.171
Land use instensity	*BCR*	0.000	0.215	0.681	0.115
	*FAR*	0.000	1.907	6.825	1.307
	*ABH*	0.000	4.839	11.426	2.493
Location	*Mdis_DC*	0.098	1.728	4.796	0.953
	*Mdis_NCC*	0.897	1077.893	3442.630	726.559
	*Mdist largepark*	0.000	23.522	68.211	10.200

**Table 3:** Global spatial autocorrelation analysis of urban vibrancy.

Vibrancy Metrics	Global Moran’s Index	*z*-Scores	*p*-Value
RPD derived vibrancy	0.444562	28.469256	0.0000
SCB derived vibrancy	0.728335	46.12009	0.0000

**Table 4:** Analysis results of multiple linear stepwise regression model.

Variables	Model 1 (RPD)	Model 2 (SCB)
*Constant*	169.248	179.849
*MU*	0.028 ** (49.422)	0.002 *** (59.722)
*Mdist_NBS*	0.000 *** (−0.183)	——
*Den_busstop*	——	0.000 *** (0.57)
*Mdis_DC*	0.009 *** (−5.306)	0.000 *** (−15.94)
*Mdist largepark*	——	0.008 *** (−0.018)
*Mdis_NCC*	0.01 *** (−23.14)	0.000 *** (−51.882)
*BCR*	——	——
*FAR*	0.000 *** (50.849)	0.000 *** (54.463)
*ABH*	0.036 ** (1.574)	——
R^2^	0.311	0.437
Adjusted R^2^	0.307	0.434

** and *** indicate statistical significance at 5% and 1% levels, respectively; numbers in parentheses represent the corresponding standard errors of the coefficients.

**Table 5:** Calculation results of geographical weighted regression (GWR) model.

GWR model	Min	Max	Median	Mean	Std	R^2^	Adjusted R^2^
Model 1: RPD derived vibrancy						0.359	0.331
Intercept	100.14782	317.36943	173.90345	184.45358	48.80974		
*MU*	−17.79050	111.54102	45.38315	39.23888	27.49579		
*Mdist_NBS*	−0.32667	0.07025	−0.22917	−0.20320	0.09976		
*Mdis_DC*	−20.82062	2.45218	−6.52870	−6.55957	4.29775		
*Mdis_NCC*	−74.04680	7.68068	−24.65114	−24.66361	12.09878		
*FAR*	27.83939	71.32490	48.93066	49.63042	9.04988		
*ABH*	−2.81929	4.37277	2.33342	1.87473	1.52163		
Model 2: SCB derived vibrancy						0.652	0.636
Intercept	−31.89531	463.52870	258.67109	227.60138	119.15618		
*MU*	−25.39452	119.09335	50.60132	49.60966	38.57372		
*Den_busstop*	0.23950	0.84073	0.60522	0.60242	0.14587		
*Mdis_DC*	−64.796852	2.677023	−27.16403	−24.46849	14.78702		
*Mdis_largepark*	−0.08134	0.04079	−0.00780	−0.01437	0.03171		
*Mdis_NCC*	−178.29393	8.48004	−69.29600	−74.75974	53.41233		
*FAR*	30.41069	94.63752	41.56988	49.83385	15.65210		

## Data Availability

The data presented in this study are available on request from the corresponding author. The data are not publicly available due to the data also forms part of an ongoing study.
